# Docking studies of flavonoid derivatives as potent HIV-1 integrase inhibitors

**DOI:** 10.1186/1471-2334-14-S3-E6

**Published:** 2014-05-27

**Authors:** N Premjanu, C Jaynthy, C Soniyagandhi

**Affiliations:** 1 Sathyabama University, Chennai, Tamilnadu, India

## Background

HIV-1 integrase is responsible for the transfer of virally encoded DNA into the host chromosome. The process of integration occurs through 3 essential steps: formation of the preintegration viral DNA complex, 3’processing and 5’ strand transfers. Provirus ancestral pol protein is a component of preintegration viral DNA complex, which has reverse transcriptase domain, Ribonuclease H like domain, integrase, N terminal and Zinc binding domain. To complete the integration process, strand transfer should take place. This work involves *in silico* prediction of the flavonoids as inhibitors of integrase activity.

## Methods

In this experiment, docking studies were performed by modeling *Pol* protein: P10266 (HERV-K_5q33.3 provirus ancestral *Pol* protein), Q9UQG0 (HERV-K_3q27.3 provirus ancestral *Pol* protein), Q9BXR3 (HERV-K_7p22.1 provirus ancestral *Pol* protein) and docking with the flavonoid compound AC1NSUMK, Quercetin 3 arabinoside and compared with Raltegravir.

## Results

Docking studies illustrate the binding between the *Pol* protein and flavonoid compound. In docking, the interactions were found in P10266 with AC1NSUMK at GLN378, GLN338, GLY283 and TYR375 with -7.71 kcal/mol. P10266 with Raltegravir at VAL369 and THR371 with -7.68 kcal/mol binding energy. The binding site resembles a saddle shape cleft which has positively charged residues.

## Conclusion

The protein along with the AC1NSUMK with four hydrogen bonds proves to be a most stable complex inhibiting the activity of integrase. The interaction shows that it binds to the protein and specifically interferes with its strand transfer activity. Hence, this may be one of the potential candidates for inhibition of HIV activity.

